# Optimal indicators for identification of compensatory sagittal balance in patients with degenerative disc disease

**DOI:** 10.1186/s12891-021-04063-5

**Published:** 2021-02-21

**Authors:** Shengbo Niu, Xiao Zhai, Yuanyuan Chen, Huan Yang, Changwei Yang, Ming Li

**Affiliations:** 1 Department of Orthopedics, Changhai Hospital, Naval Medical University, Shanghai, China; 2 Reproductive Medicine Center, Changhai Hospital, Naval Medical University, Shanghai, China

**Keywords:** Optimal indicators, Compensatory sagittal balance, Degenerative disc disease, Compensatory mechanism, Sacral slope, Pelvic tilt

## Abstract

**Study design:**

A retrospective study.

**Background:**

To determine whether radiological parameters such as maximal lumbar lordosis-maximal thoracic kyphosis (maxLL-maxTK), sacral slope-pelvic tilt(SS-PT) and sacral slope/pelvic tilt (SS/PT) could be used as indicators for the diagnosis of degenerative disc disease (DDD) in compensatory sagittal balanced patients.

**Methods:**

Medical records of sagittal balanced DDD patients and asymptomatic adults within our hospital registry from July 2019 to November 2019 were reviewed. General characteristics and radiological parameters were evaluated between the two groups. Analysis of covariance with age as a covariate was conducted, followed by receiver operating characteristic (ROC) analysis and areas under the curve (AUC) calculation. The max Youden index was calculated to identify the optimal sensitivity specificity pairs.

**Results:**

A total of 42 DDD patients and 199 asymptomatic adults were included. For those parameters that showed significant differences between the two groups, AUC for SS/PT and SS-PT were the largest, reaching 0.919 and 0.936, respectively. The sensitivity was 0.749, the specificity was 0.952 and the max Youden index was 0.701 when SS/PT = 1.635 was used as threshold. The max Youden index was found for a threshold of SS-PT =8.500, for which the sensitivity increased to 0.854, while the specificity decreased to 0.857.

**Conclusions:**

Both SS/PT and SS-PT were significantly different between sagittal balanced DDD patients and asymptomatic adults. SS/PT < 1.6 and SS-PT < 8.5 could be used as indicators for the diagnosis of DDD patients with compensatory sagittal balance.

## Background

**Fig. 1:**
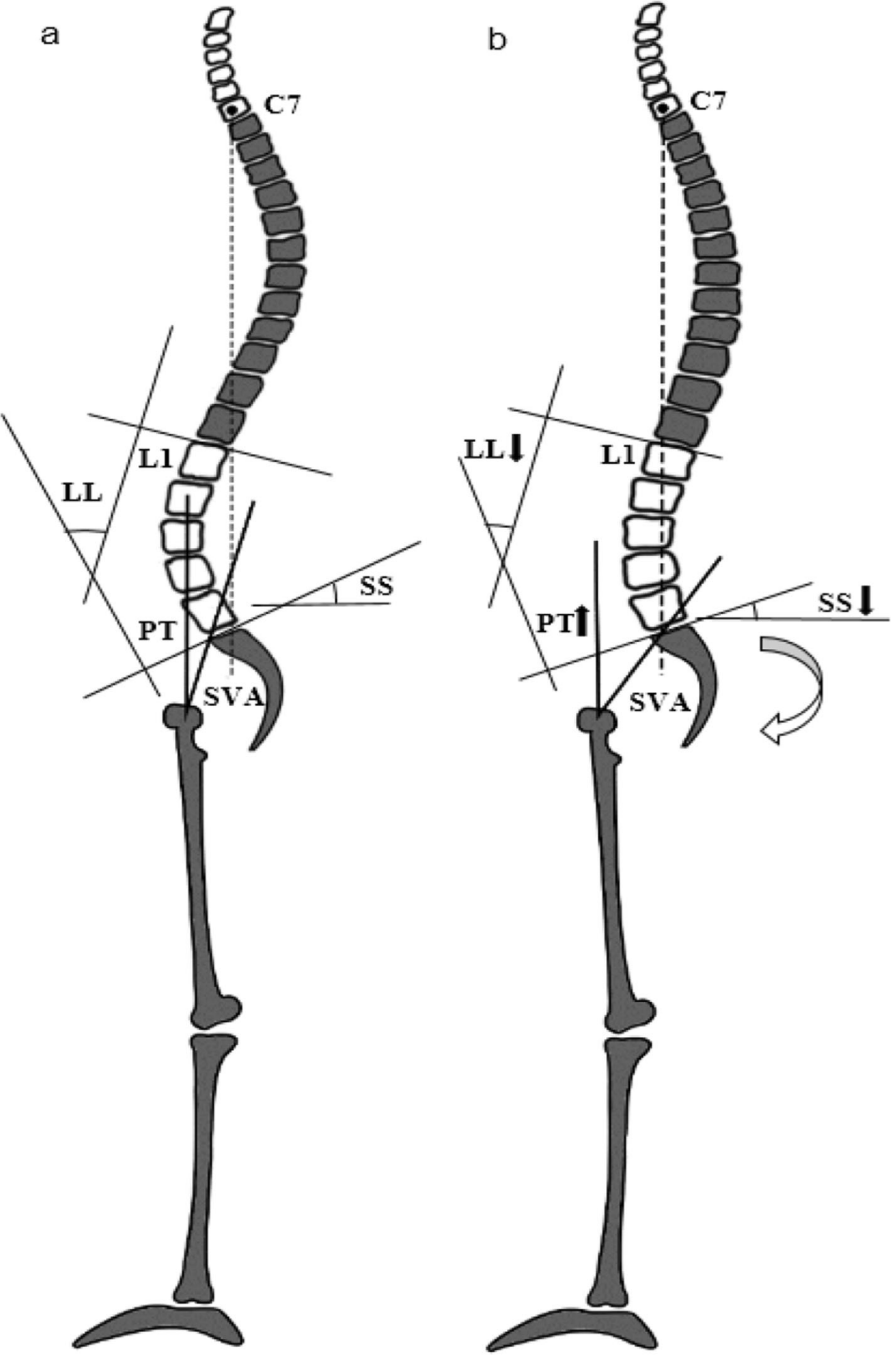
**a** Asymptomatic adult with sagittal balance: LL, SS, PT, SVA are in the normal range and the pelvis is in the neutral position; **b** DDD patients with compensatory sagittal balance: LL decreases causing SVA to move forward, the pelvis rotates backwards, SS decreases and PT increases, the femoral head is forward, the sacrum and the spine are backward. This causes SVA to stay behind the perpendicular line passing through the middle of the femoral head. (LL, lumbar lordosis; SS, sacral slope; PT, pelvic tilt; SVA, sagittal vertical axis; DDD, degenerative disc diseases)

Intervertebral discs degeneration is a normal process of aging that can be accelerated by different environmental and biological factors. It associated with pain, referred to as degenerative disc diseases (DDD) [1]. An ideal system of classification among types of DDD does not currently exist [2]. In general, DDD is a spectrum of diseases, which may present as disc herniation, spinal stenosis, spondylolisthesis, facet joint arthropathy, or their combination. Recent studies suggest that sagittal balance is important in DDD in which it closely associated with the patient’s quality of life (QOL) [3, 4]. The Roussouly classification of sagittal profiles, including Type 1, Type 2, Type 3 and Type 4, can help to discover the association between spinal balance and the development of degenerative changes in the spine [5]. T1(first thoracic vertebrae) tilt, thoracic kyphosis (TK), lumbar lordosis (LL), sacrum slope (SS), pelvic tilt (PT), pelvic incidence (PI) and sagittal vertical axis (SVA) have been identified as important spinopelvic parameters in maintaining spinal sagittal balance [6], and proven to be essential and effective reference in spinal fusion surgery [3]. Many studies have reported a significant loss of LL in patients with DDD, especially after spinal fusion surgery, resulting in a compensative increase in PT [7–9]. Increasing PT during standing posture was reported to reflect patients need to compensate for their proximal spinal deformity [10], and degenerative loss of lordosis moves the spine forward, as a result, compensatory mechanisms such as pelvic retroversion and knee flexion lead to posterior pelvic shift [11] . The backward rotation of the pelvis can continue to a certain extent, the femoral head is forward result from the increasing tilt of the pelvis, meanwhile, the sacrum and the spine are backward. This causes the C7(seventh cervical vertebrae) plumb line to stay behind the perpendicular line passing through the middle of the femoral head, and the gravity line to fall between the feet (Fig. 1). As a result, The full body is in an uneconomic compensatory balance because the maintenance of this balance increases the tension of the posterior spinal muscle causing energy-consuming and low back pain.

Schwab et al. [12] find a negative correlation between PT (threshold 20°) and health-related QOL. So PT < 20° was incorporated into an adult spinal deformity classification. However, a high absolute value of PT does not necessarily represent increased retroversion, PT > 20° can also be an anatomic trait and simply reflect a high PI, in a similar way, the PT threshold may need to be less than 20° for patients with a low PI [13] . Reciprocal relationships between the SS and the characteristics of the lumbar curvature are considered an essential component of overall sagittal alignment. The SS of Type 1 and Type 2 are less than 35°, which are usually associated with a low PI indicating a relatively less compensatory capacity of sagittal balance than Type 3 and Type 4 [5]. Futher more, the normal range of those spinopelvic parameters are so wide due to individual differences [14] and measurement errors that it is challenging to differentiate the DDD patiens with compensatory sagittal balance from those with normal sagittal balance by using a single radiographic parameter alone. DDD resulting in loss of lumbar lordosis, can lead to sagittal plane deformities [15]. The loss of lumbar lordosis can be considered as the initiating event of sagittal imbalance [16]. Because of the potential mechanism of compensation, just like pelvic retroflexion, thoracolumbar hyperextension, knee flexion, ankle flexion, and finally cervical extension [17], in the DDD patients with SVA ≤ 50 mm, some present with a compensatory sagittal balance, while others are still in normal sagittal balance. We believe that the loss of lumbar lordosis to the initiation of compensatory mechanism is a gradual process. Difficulties in distinguishing between those two groups in DDD patients with sagittal balance not only influence the indication for spinal surgery [18], but also conceal the severity of the disease and mislead the treatment strategy. The recognition of compensatory sagittal balance has some clinical significance for the diagnosis and treatment of DDD. Nevertheless, few parameters have been used in the assessment of a state of compensatory sagittal balance, and comparison of these parameters between DDD patients with sagittal balance and asymptomatic adults without DDD has not been extensively explored. The purpose of this study was to explore possible correlations between the composite radiological parameters which could reduce their individual differences to some extent such as maxLL-maxTK, SS-PT, SS/PT and the diagnosis of DDD patients with compensatory sagittal balance, and identify which parameters could be used to discriminate DDD patients with compensatory sagittal balance from those in normal sagittal balance.

## Methods

### Patient selection

Adult patients (age ≥ 18 years) with sagittal balanced DDD including lumbar disc herniation (LDH), lumbar spinal stenosis (LSS), and with or without instability who scheduled for spine surgery in our hospital between July 2019 and November 2019 and met the inclusion and exclusion criteria were retrospectively reviewed as DDD group. The inclusion criteria were: 1) a minimum 3-month history of low back pain and/or lower limb pain and/or lower limb numbness; 2) DDD grade 3 according to the Schneiderman classification on MRI showing a hypointense nucleus with disc space narrowing on at least one level [19]; 3) sagittal balanced patients (SVA ≤ 50 mm) [20]. The exclusion criteria were: 1) patients with red flag symptoms such as scoliosis, trauma or fracture, ankylosing spondylitis, osteoporosis, pregnancy, or tumor; 2) patients with a previous surgery history of spine or artificial femoral head replacement. Asymptomatic adults (age ≥ 18 years) whose routine physical examination were conducted in our hospital in the same time were free of spinal disease and used as the control (normal) group. The exclusion criteria were as follows: 1) a history of low back pain and/or lower limb pain and/or lower limb numbness; 2) subjects with conditions such as trauma or fracture, osteoporosis, pregnancy, tumor, a previous surgery history of spine or artificial femoral head replacement. The radiographic imaging of the full-length spine were taken in the standard, natural and comfortable upright position in both cohorts, and those showing horizontal displacement of two femoral heads or irregular superior endplate of the first sacral vertebrae or other conditions in which the parameter measurement cannot be performed were excluded. This study was approved by the Institutional Review Board of our university, and all subjects involved provided written informed consent.

### Data collection

**Fig. 2:**
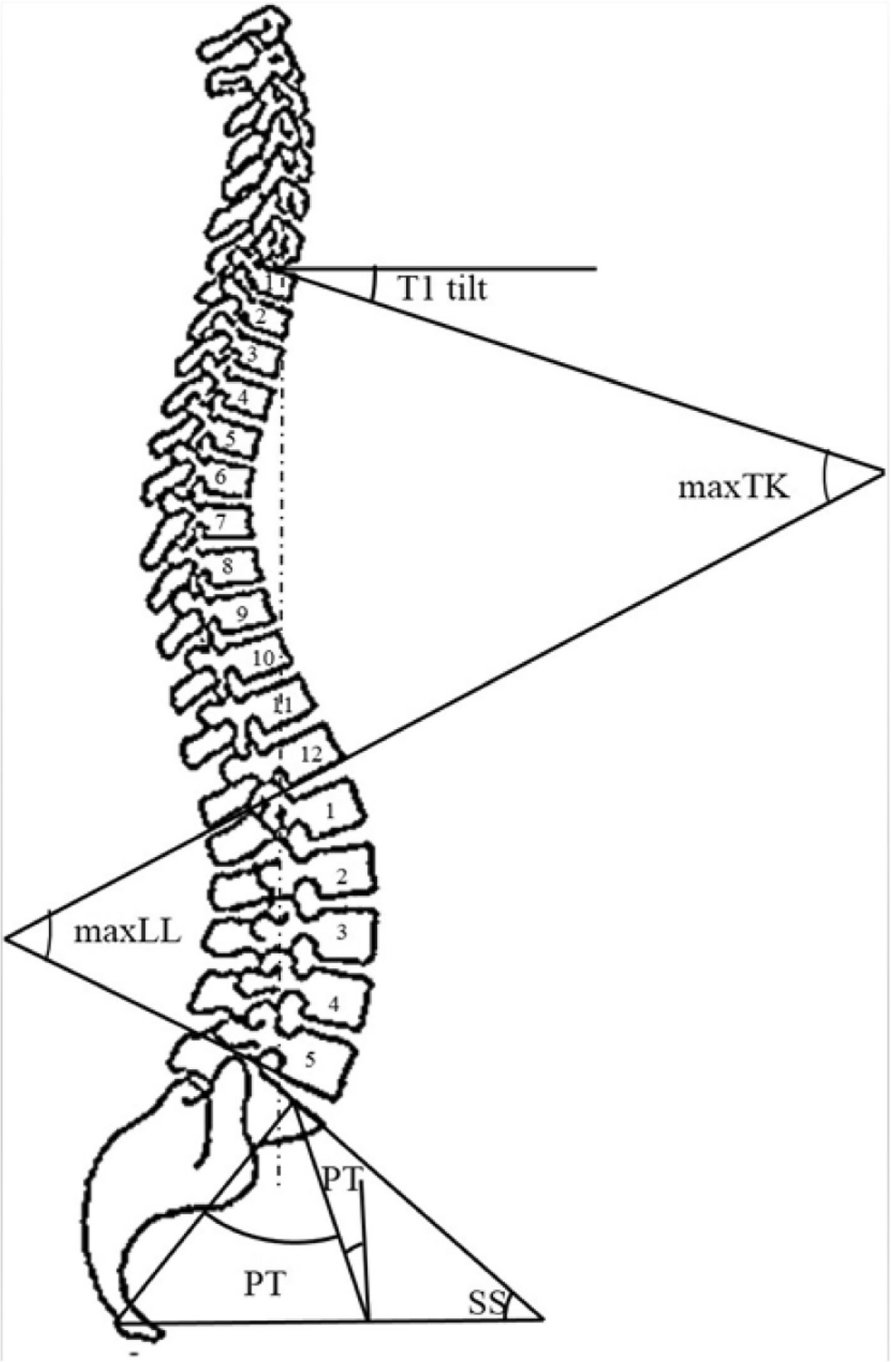
Radiographic parameters measured on the standing full-spine lateral radiographs (T1 tilt; maxTK, maximal thoracic kphyosis; maxLL, maximal lumbar lordosis; SS, sacral slope; PT, pelvic tilt; and PI, pelvic incidence; SVA, sagittal vertical axis)

General characteristics of all subjects including age, gender, body mass index (BMI) and the demographic breakdown of highly prevalent conditions such as hyperlipidemia, hypertension, diabetes mellitus, peripheral vascular disease (PVD), chronic obstructive pulmonary disease (COPD), smoking, drinking, depression were collected. Radiographic parameters were measured on the standing full-spine lateral radiographs and evaluated (Fig. 2), including the angle between the horizontal and superior endplate of T1 (T1 tilt), the angle between the superior endplate of T1 and the inferior endplate of T12 using the Cobb method (T1–12 kyphosis, maxTK), the angle between the superior endplate of L1 and the superior endplate of S1 using the Cobb method (L1-S1 lumbar lordosis, maxLL) [21], the angle formed by a line drawn along the endplate of the sacrum and a horizontal reference line (sacral slope, SS), the angle formed by a line drawn from the midpoint of the sacral endplate to the center of the bicoxofemoral axis and vertical plumb line (pelvic tilt, PT), the angle formed by a line drawn between the center of the femoral head and the sacral endplate (pelvic incidence, PI) and the distance between the C7 plumb line and the posterior corner of the sacrum (sagittal vertical axis, SVA) [17, 22, 23].

The relationship between maxLL and maxTK was expressed as maxLL-maxTK (maxLL minus maxTK). The relationship between SS and PT was expressed as SS-PT and SS/PT (SS minus PT and SS divided by PT). The relationship between LL and PI was expressed as LL-PI (LL minus PI). Two fellowship trained orthopaedic spine surgeons with at least 2 years working experience measured all the radiographic parameters independently, and the intraclass correlation coefficients (ICC) of the radiographic parameters between them are as follows: T1 tilt (0.92, *p* < 0.01), maxTK (0.84, *p* < 0.01), maxLL (0.85, *p* < 0.01), SS (0.85, *p* < 0.01), PT (0.85, *p* < 0.01), PI (0.91, *p* < 0.01), SVA (0.90, *p* < 0.01). So the mean values of the radiographic parameters messured by them were used as final results for analysis. All the radiological data mentioned above were compared between the DDD group and the normal group. To identify the most important index for predicting the diagnosis of DDD with compensatory sagittal balance, ROC analysis was also performed and the max Youden index was calculated.

### Statistical analysis

We performed statistical analyses using Statistic Package for Social Science (SPSS) 22.0 statistics software (SPSS Inc., Chicago, IL), and listed measurement data in the form of mean and standard deviation (SD), enumeration data in terms of ratio. Chi-square test was used for enumeration data comparison between the DDD group and the normal group. Independent two-sample t-test and Wilcoxon rank sum test were used to compare the differences of measurement data between the two groups. The general characteristics which were different between the two groups(*P* < 0.05) were used as a covariate to perform comparisons between radiological parameters and the level of significance was set at *p* < 0.05. For each parameter, diagnostic screening and confirmatory tests were performed by calculating true positive, true negative, false positive, false negative values, sensitivity and specificity of different cut-off points were calculated. ROC analysis was performed and AUC were calculated for radiological parameters to see which one had high discrimination performance with AUC being greater than 0.9 for the DDD with compensatory sagittal balance. The maximal Youden index (maxYI) of each parameter was also calculated. The cut-off point corresponding to it was taken as the threshold value that determine the optimal sensitivity-specificity pair.

## Results

A total of 42 DDD patients and 199 asymptomatic adults were included in the DDD group and the control (normal) group, respectively. There were no significant differences in PI and the general characteristics with the exception of age between two groups, while age and other residual parameters were significantly different between two groups (Table 1). The analysis of covariance using age as a covariate showed no significant differences in T1 tilt, maxTK and PI between the twogroups, while significant differences were found in maxLL, maxLL-maxTK, SS, PT, SS/PT, SS-PT, maxLL-PI, SVA (Table 2).

**Table 1:** General characteristics of subjects in two groups

Characteristics	DDD group(42)	Normal group(199)	*P* value
Age (years)	58.88 ± 11.93	42.78 ± 15.88	< 0.001
BMI (kg/m^2^)	22.59 ± 4.76	23.21 ± 3.91	0.146
Gender(%)	–	–	0.238
Male	33.3	43.2	–
Female	66.7	56.8	–
Hyperlipidemia (%)	–	–	0.394
Yes	31.0	24.6	–
No	69.0	75.4	–
Hypertension (%)	–	–	0.192
Yes	40.5	30.2	–
No	59.5	69.8	–
Diabetes (%)	–	–	0.329
Yes	28.6	21.6	–
No	71.4	78.4	–
PVD(%)	–	–	0.762
Yes	14.3	12.6	–
No	85.7	87.4	–
COPD(%)	–	–	0.286
Yes	23.8	16.8	–
No	76.2	83.2	–
Smoking(%)	–	–	0.250
Yes	19.0	12.3	–
No	81.0	87.7	–
Drinking(%)	–	–	0.346
Yes	31.0	38.7	–
No	69.0	61.3	–
Depression	0	0	–

**Table 2:** Before and after covariance analysis with age as covariate of radiographic parameters

Parameters	DDD group(42)	Normal group(199)	*P* value	
			before	after
T1 tilt(°)	22.90 ± 6.34	19.73 ± 6.08	0.003	0.065
maxTK(°)	40.10 ± 11.25	36.15 ± 9.19	0.016	0.411
maxLL(°)	41.43 ± 9.80	49.50 ± 9.49	< 0.001	< 0.001
maxLL-maxTK(°)	1.33 ± 8.75	13.35 ± 9.22	< 0.001	< 0.001
SS(°)	24.14 ± 7.03	33.22 ± 7.40	< 0.001	< 0.001
PT(°)	24.19 ± 6.81	14.34 ± 7.15	< 0.001	< 0.001
PI(°)	48.33 ± 11.17	47.56 ± 10.56	0.669	0.822
SS/PT(°)	1.06 ± 0.36	3.04 ± 3.62	< 0.001	0.001
SS-PT(°)	−0.05 ± 8.18	18.88 ± 10.02	< 0.001	< 0.001
LL-PI(°)	−6.90 ± 9.76	1.94 ± 9.90	< 0.001	< 0.001
SVA (mm)	20.14 ± 20.84	2.4 ± 22.00	< 0.001	0.001

**Fig. 3:**
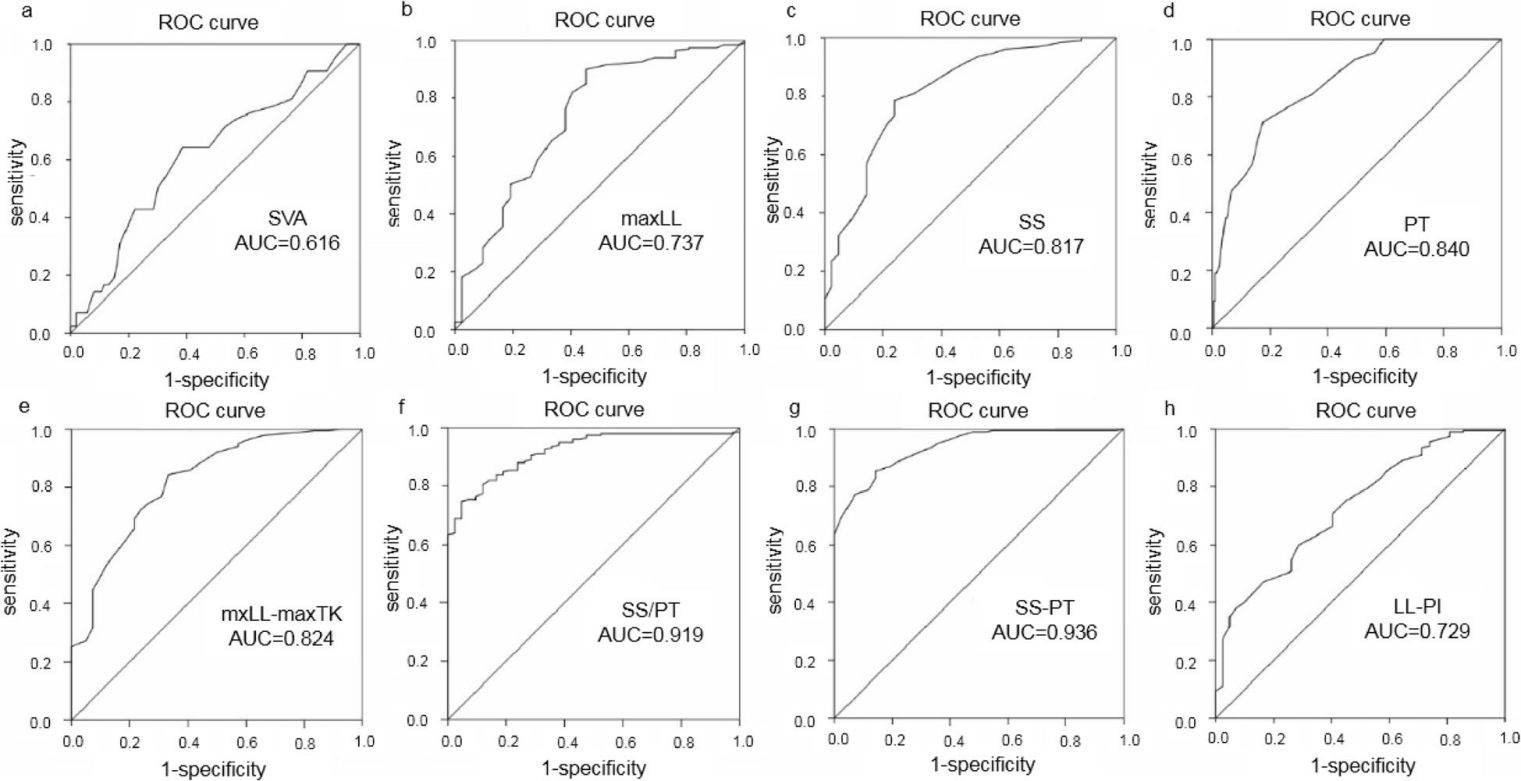
Prediction of DDD patients with compensatory sagittal balance by receiver operating characteristic (ROC) curves of radiological parameters. Areas under the curves (AUC) were 0.616 for maxTK (**a**), 0.737 for maxLL (**b**), 0.817 for SS (**c**), 0.840 for PT (**d**), 0.824 for maxLL-maxTK (**e**), 0.919 for SS/PT (**f**), 0.936 for SS–PT (**g**), 0.729 for LL–PL (**h**) (DDD, degenerative disc diseases; max TK, max thoracic kyphosis; max LL, max lumbar lordosis; SS, sacrum slope; PT, pelvic tilt; PI, pelvic incidence)

**Fig. 4:**
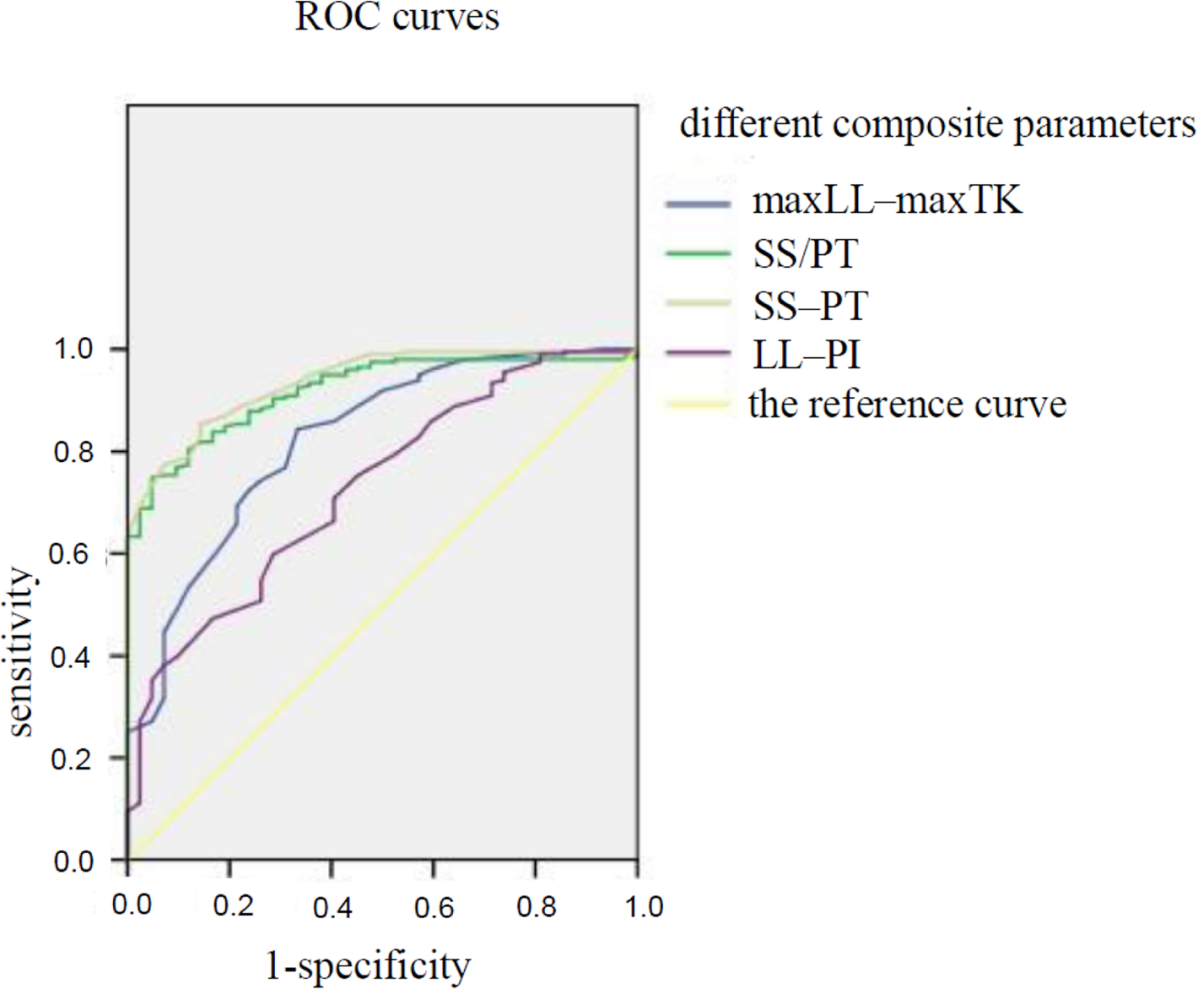
Comparison of areas under the curves (AUC) of different composite parameters. The results showed SS–PT and SS/PT had the largest AUC indicating high diagnostic value

Power analysis and sample size (PASS) 15 was used to calculate the sample size. A sample of 18 from the positive group and 199 from the negative group achieves 89% power to detect a difference of 0.2000 between the AUC under the null hypothesis of 0.7000 and an AUC under the alternative hypothesis of 0.9000 using a two-sided z-test at a significance level of 0.050. The data are continuous responses. The AUC is computed between false positive rates of 0.00 and 1.00. The ratio of the standard deviation of the responses in the negative group to the standard deviation of the responses in the positive group is 1.00. So the sample of 42 from the positive group is enough to determine statistical significance between the two cohorts analyzed. It was found that the AUC for the composite indexes such as SS-PT and SS/PT were the largest, reaching 0.919 and 0.936 respectively (as shown in Figs. 3 and 4). The optimal sensitivity-specificity pair was obtained, showing that the sensitivity was 0.749, the specificity was 0.952 and the maxYI was 0.701 when SS/PT = 1.635 was used as the threshold. The maxYI was found for a threshold of SS-PT = 8.500, when the sensitivity increased to 0.854, and the specificity decreased to 0.857.

## Discussion

The aim of the present study was to find potential radiological parameters, especially the composite indexes, that could be used to distinguish between DDD patients with compensatory sagittal balance and DDD patients with normal sagittal balance. It was found in previous studies that age was associated with the occurrence of DDD [24] and the mean age was different between the two groups(*P* < 0.05). Therefore we used age as a covariate to perform analysis of covariance. Finally, we found that two composite indexes SS/PT, SS-PT and some other parameters were significantly related to the DDD patients with compensatory sagittal balance. The analysis of ROC curves showed that both SS/PT and SS-PT had larger AUCs and could well tell compensatory sagittal balanced patients with DDD from normal sagittal balanced patients with DDD. We set SS/PT > 1.6 and SS-PT > 8.5 as indicators for normal sagittal balanced patients with DDD, and SS/PT < 1.6 and SS-PT < 8.5 as indicators for the diagnosis of DDD patients with compensatory sagittal balance. Previous studies used to address the significant correlation between sagittal parameters and health-related QOL [25]. In this study, we introduced almost all common spinopelvic sagittal parameters, including morphological parameters (TK, LL and PI), position parameters (SS and PT) [26], and used maxTK and maxLL as representative of the global sagittal alignment of thoracic kyphosis and lumbar lordosis. Since these single parameters were of great variability during different postures and measurement, we alternatively introduced composite indexes of maxLL-maxTK, SS/PT, SS-PT and LL-PI.

The most important results found in this study are that decreased SS/PT and SS-PT are significantly correlated with the compensatory sagittal balance of DDD patients. DDD patients with compensatory sagittal balance are characterized by decreased SS and increased PT as demonstrated in our illustrated cases in Table 2, which also indicates the important role of the compensatory mechanism of the pelvis in maintaining sagittal balance. SS and PT are thought to be associated with the compensatory mechanism in the pelvic area which is the keystone of equilibrium of the human body and gravity line [27]. During the compensatory process, as previously mentioned, the pelvis rotate around the femoral heads following the bicoxo-femoral axis, which is similar to that during hip extension. Due to the contraction of the hip extensor muscles, this motion of hip extension results in posterior positioning of the sacrum related to the bicoxo-femoral heads and increasing the sacro-femoral distance (Fig. 1). The possibility of rotation of the pelvis around the bicoxo-femoral axis is one of the most important compensatory mechanisms of sagittal balance. This mechanism permits to compensate for the anterior translation of the axis of gravity [7]. A low SS means a low ability of pelvic tilting, conversely, a high SS means a higher possibilities of retroversion and when the pelvis rotates backward (retroversion), PT increases; when the pelvis rotates forward (anteversion), PT decreases [26]. Because of SS cannot be a negative value, a high PI have a much wider range for retroversion [15]. Based on what was mentioned above, we conclude that both SS/PT and SS-PT can be as a pelvic regional sagittal alignment of DDD patients with compensatory sagittal balance. The loss of lumbar lordosis can be considered as the initiating event of sagittal imbalance [16]. This loss of the normal lordosis pushes the C7 plumb line forward [11]. Then the pelvic compensatory mechanism is activated, and SS/PT < 1.6, SS-PT < 8.5 can be as indicators of DDD patients with compensatory sagittal balance according to this study. During the spinal surgery, LL should not be studied as a single curve and there is a strong correlation between SS and LL [28]. To obtain an ideal state of other indexes, efforts should be made to maintain a normal LL, in other words, high incidence high lordosis and low incidence low lordosis [9]. Similarly, DDD patients present with a compensatory sagittal balance need a higher lumbar lordosis comparing to those in normal sagittal balance according to this study.

This study had some limitations that should be addressed. First, our study was a single-center study and the sample size was relatively small. However, the number (42) of DDD patients meets the minimum number (18) to find statistical significance by a power analysis. Second, additional global sagittal alignment markers that take PT into account such as TPA(T1 pelvic angle) were not studied in this research. They are well worth studying in a follow-up study. Third, according to Roussouly classification, Type 2 and Type 4 don’t have the same capacity of compensation because the SS of Type 1 and Type 2 are less than 35°, which are usually associated with a low PI indicating a relatively less compensatory capacity of sagittal balance than Type 3 and Type 4. However, the Roussouly classification is not introduced in the description of the cohorts both because Roussouly classification which is based asymptomatic people with the average age of 27 years might not be very precise for DDD patients and the purpose of this study was to determine radiological parameters which could be used as indicators for the diagnosis of DDD patients with compensatory sagittal balance. Fourth, the subjects in two groups were not matched to the age factor and since degeneration of the intervertebral disc naturally occur in older people, it is difficult to match well. Alternatively, we performed analysis of covariance with age as a covariate to reduce the bias. Therefore, large-scaled and multicenter studies should be performed to make a more comprehensive investigation into the effectiveness of SS/PT and SS-PT in assessing compensatory sagittal balance in DDD patients.

## Conclusions

Both SS/PT and SS-PT were significantly different between compensatory sagittal balanced DDD patients and asymptomatic adults. SS/PT < 1.6 and SS-PT < 8.5 could be used as indicators for the diagnosis of DDD patients with compensatory sagittal balance.

## Data Availability

The datasets used or analyzed during the study are available from the corresponding author on reasonable request.

## Abbreviations

maxLL-maxTK: Maximal lumbar lordosis-maximal thoracic kyphosis
SS-PT: Sacral slope-pelvic tilt
SS/PT: Sacral slope/pelvic tilt
DDD: Degenerative disc diseases
ROC: Receiver operating characteristic
AUC: Areas under the curve
QOL: Quality of life
T1: First thoracic vertebrae
TK: Thoracic kyphosis
LL: Lumbar lordosis
SS: Sacrum slope
PT: Pelvic tilt
PI: Pelvic incidence
SVA: Sagittal vertical axis
C7: Seventh cervical vertebrae
LDH: Lumbar disc herniation
LSS: Lumbar spinal stenosis
BMI: Body mass index
PVD: Peripheral vascular disease
COPD: Chronic obstructive pulmonary disease
maxTK: T1–12 kyphosis
maxLL: L1-S1 lumbar lordosis
LL-PI: Lumbar lordosis-pelvic incidence
ICC: Intraclass correlation coefficients
SPSS: Statistic package for social science
SD: Standard deviation
maxYI: Maximal Youden index
PASS: Power analysis and sample size
TPA: T1 pelvic angle
